# Molecular and Imaging Biomarkers in Alzheimer’s Disease: A Focus on Recent Insights

**DOI:** 10.3390/jpm10030061

**Published:** 2020-07-10

**Authors:** Chiara Villa, Marialuisa Lavitrano, Elena Salvatore, Romina Combi

**Affiliations:** 1 School of Medicine and Surgery, University of Milano-Bicocca, 20900 Monza, Italy; 2 Institute for the Experimental Endocrinology and Oncology, National Research Council (IEOS-CNR), 80131 Naples, Italy; m.lavitrano@ieos.cnr.it; 3 Department of Neurosciences and Reproductive and Odontostomatological Sciences, Federico II University, 80131 Naples, Italy; elena.salvatore@unina.it

**Keywords:** Alzheimer’s disease, biomarker, amyloid beta, neuroimaging, cerebrospinal fluid

## Abstract

Alzheimer’s disease (AD) is the most common neurodegenerative disease among the elderly, affecting millions of people worldwide and clinically characterized by a progressive and irreversible cognitive decline. The rapid increase in the incidence of AD highlights the need for an easy, efficient and accurate diagnosis of the disease in its initial stages in order to halt or delay the progression. The currently used diagnostic methods rely on measures of amyloid-β (Aβ), phosphorylated (p-tau) and total tau (t-tau) protein levels in the cerebrospinal fluid (CSF) aided by advanced neuroimaging techniques like positron emission tomography (PET) and magnetic resonance imaging (MRI). However, the invasiveness of these procedures and the high cost restrict their utilization. Hence, biomarkers from biological fluids obtained using non-invasive methods and novel neuroimaging approaches provide an attractive alternative for the early diagnosis of AD. Such biomarkers may also be helpful for better understanding of the molecular mechanisms underlying the disease, allowing differential diagnosis or at least prolonging the pre-symptomatic stage in patients suffering from AD. Herein, we discuss the advantages and limits of the conventional biomarkers as well as recent promising candidates from alternative body fluids and new imaging techniques.

## 1. Introduction

**Figure 1:**
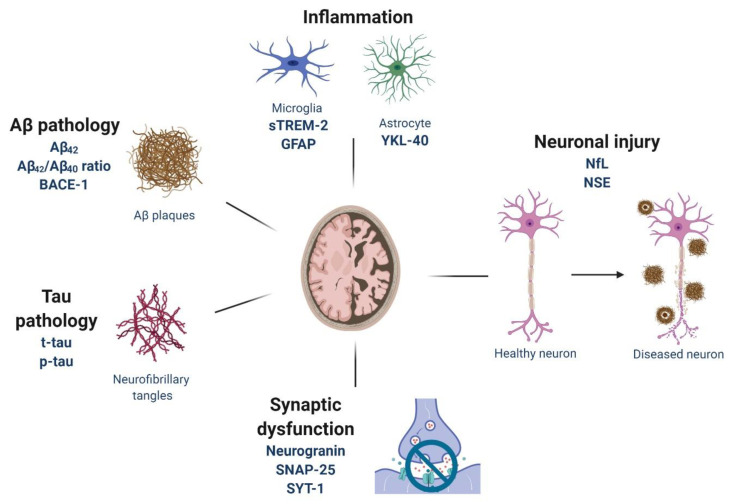
Main pathological mechanisms occurring in Alzheimer’s disease and their associated fluid biomarkers.

Alzheimer’s disease (AD) represents the most common form of dementia in the elderly population worldwide, accounting for up to 80% of all diagnoses [1]. AD is clinically characterized by irreversible and progressive neurodegeneration leading to memory deterioration, behavioral changes and cognitive dysfunction, resulting in autonomy loss, which ultimately requires full-time medical care [2]. The neuropathological hallmarks include the presence of extracellular senile plaques constituted by the amyloid-β (Aβ) peptides and intracellular neurofibrillary tangles (NFTs) consisting of hyper-phosphorylated paired helical filaments (PHFs) of the microtubule-associated protein tau (MAPT) [3]. Aβ plaques are composed of various Aβ peptides, including the 40 and 42 amino acid products (Aβ_40_ and Aβ_42_), generated as a result of the sequential proteolytic cleavage of Aβ precursor protein (APP) by β-site APP-cleaving enzyme 1 (BACE-1) and the γ-secretase complex [4]. During the early stage of the disease, extraneuronal Aβ deposits, intraneuronal NFTs and neuritic threads are found in the entorhinal cortex and in the hippocampus, which are the key regions of memory and learning functions. However, in addition to Aβ and tau pathology, other processes, such as synaptic dysfunctions and microglia-mediated inflammation also play an important role in AD pathogenesis and may correlate with cognitive decline (Figure 1) [5,6].

Most AD cases are sporadic with a late onset, usually occurring in individuals aged 65 or older, and the main risk factors are aging and carrying the ε4 allele of Apolipoprotein E (ApoE) [7]. Conversely, the rare early-onset forms of AD affect individuals under 65 years of age and show an autosomal dominant pattern of inheritance, generally presenting a positive family history. These patients carry mutations in one of the known genes, namely *PSEN1*, *PSEN2* and *APP*, encoding the presenilin-1, presenilin-2 and APP proteins, respectively. All of them are involved in the maturation and processing of APP, leading to an increased production or aggregation of Aβ peptide [8].

Although behavioral symptoms can be alleviated by actual therapeutic strategies, drugs that prevent or halt the disease course are still not available [9]. The lack of success of disease-modifying therapy may be partially explained by the complex etiology in its pathophysiology and the limitations in past clinical trials designed on enrolled participants with mild-to-moderate AD or with no Aβ pathology [10]. In this regard, a biomarker holds promise for enabling more effective drug development in AD and establishing a more personalized medicine approach [11]. It would be a suitable indicator of the stage of disease progression, treatment monitoring and a valuable tool for epidemiological and therapeutic research. According to the latest guidelines of the National Institute on Aging and Alzheimer’s Association (NIA-AA), the recommendations for the diagnosis of pre-clinical, mild cognitive impairment (MCI) and AD dementia have been updated, unifying biological markers and imaging into AT(N) groups. This novel classification summarizes biomarkers into three categories: Aβ deposition (A), pathological fibrillary tau (T) and neurodegeneration (N) [12,13]. Currently, group A includes low levels of Aβ_42_ in the cerebrospinal fluid (CSF) or Aβ positron emission tomography (PET) ligand binding; group T includes elevated levels of CSF phosphorylated tau at threonine 181 (p-tau) and tau PET ligand binding, whereas group N includes elevated CSF total tau (t-tau), fluorodeoxyglucose (FDG)-PET hypometabolism and atrophy on magnetic resonance imaging (MRI) [13]. Increasing efforts have been made in recent years to detect biomarkers in more accessible biological matrices; therefore, in this review we discuss the clinical relevance of emerging candidate biomarkers in CSF and in other promising alternative non-invasive biological fluids as well as novel approaches in “dry” biomarkers like neuroimaging or neurophysiological techniques.

## 2. Emerging AD Biomarkers in Biological Fluids

### 2.1. Invasive CSF Biomarkers

Despite its invasiveness of collection, CSF still represents the most reliable biological fluid for biomarker detection of the central nervous system (CNS) disorders, allowing the most accurate elucidation about the molecular processes occurring during neurodegeneration. Compared with blood, CSF has the advantage of its proximity to the brain parenchyma and that it contains brain proteins which are directly secreted from the brain extracellular space.

In addition to the well-established core AD CSF biomarkers like Aβ and tau proteins, a number of candidate molecules have been investigated as potential AD biomarker, mainly related with pathological mechanisms or to other aspects of the disease pathophysiology, such as enzymatic deficits, degrading pathway, biochemical modifications or clearance. Currently, one of the most studied biomarkers is neurofilament light chain (NfL), a scaffold protein found in the neuronal cytoskeleton. After axonal injury, intracellular NfL is released in the extracellular space, leading to an increased concentration in the CSF. Therefore, it represents a non-specific marker for neuronal damage and has been largely studied in the context of neurodegenerative diseases, including multiple sclerosis (MS), Parkinson’s disease (PD), frontotemporal dementia (FTD) and amyotrophic lateral sclerosis (ALS) [14]. Elevated levels of CSF NfL were found in patients with MCI and AD, associating with the severity of memory impairment as a marker of disease progression [15,16,17]. Altogether, studies reported a very good performance of CSF NfL to distinguish AD cases from cognitively healthy controls with no evidence of structural brain damage [18]. However, the currently available evidence does not support the ability of CSF NfL to differentiate AD from disease mimics or MCI [14].

Similarly, also the presence of neuron-specific enolase (NSE) in CSF represents a marker of neuronal damage. NSE is a glycolytic enzyme involved in neuronal energy metabolism, axoplasmatic transport and cell survival. It is physiologically not secreted in the extracellular space, so elevated CSF NSE levels are regarded as the result of an upregulation of neuronal metabolic activity that follows increased energy demand. Significantly higher protein levels were found in AD patients, and alone or in combination with t-tau and p-tau, NSE further showed both high specificity and sensitivity to distinguish AD cases from healthy controls, suggesting a clinical application of this potential biomarker [19]. However, NSE could not discriminate AD from other forms of dementia [20].

The post-synaptic protein neurogranin, which is exclusively expressed in the cortex and hippocampus by excitatory neurons, seems to be a promising biomarker candidate. It is known to play an important role in learning and memory by maintaining long-term potentiation and synaptic plasticity. Neurogranin expression is highest in cortical areas, but its levels are markedly low in the frontal cortex and the hippocampus, indicating that the measurement of neurogranin in CSF could serve as a biomarker for synaptic degeneration and dendritic instability [21]. Synapse loss is a downstream effect of amyloidosis, tauopathy, inflammation and other pathological mechanisms occurring in AD and strongly correlates with decline in cognitive performance. High CSF levels of neurogranin in AD and prodromal AD have been confirmed by several studies using immunoassay recognizing both the full-length protein and the fragment peptides [22,23,24,25]. Moreover, encouraging data showed that increased neurogranin fragments in CSF correlate with cognitive decline, hippocampal atrophy measured by MRI and reduced glucose metabolism on FDG-PET [22,26]. Interestingly, the increase in CSF neurogranin seems to be specific for AD and not found in other neurodegenerative disorders, including FTD, PD, Lewy body dementia (LBD), progressive supranuclear palsy or multiple system atrophy [27].

Additional synaptic proteins, including synaptosomal-associated protein 25 (SNAP-25) and synaptotagmin-1 (SYT-1), also showed promising results as CSF biomarkers for synaptic damage and loss. Whereas SNAP-25 is found at synaptic vesicles, SYT-1 is located in the pre-synaptic plasma membrane and is essential for synaptic vesicle exocytosis and therefore neurotransmitter release [23]. The levels of both SNAP-25 and SYT-1 are decreased in the cortical areas of AD brain, reflecting the synaptic loss and degeneration occurring in AD [28,29]. Interestingly, a marked increase in both SNAP-25 and STY-1 levels in CSF was found in patients with AD or MCI as compared with controls [28,30,31]. Although these results need validation in further studies, they may represent a valuable tool regarding the relevance of synaptic degeneration and loss in AD pathogenesis and also in the clinical evaluation of patients.

Recent research proposed markers of glial activation as potential biomarkers for AD. Among them, one of the most promising is the triggering receptor expressed on myeloid cells 2 (TREM-2), mostly because there is a strong genetic association between TREM-2 and AD. TREM-2 play several roles in microglia, including cytokine release, proliferation, APOE binding and shielding of Aβ plaques [32,33]. It is a transmembrane protein and its soluble domain (sTREM-2) is released into the extracellular space and can be measured in both CSF and blood. The majority of studies reported increased levels of sTREM-2 in AD vs. controls which dynamically change during the disease course, reaching the peak in the later asymptomatic stage and early symptomatic phase of late-onset AD or in the genetic forms of AD [34,35,36,37]. However, CSF sTREM-2 is closely associated with tau-related neurodegeneration but not with Aβ pathology [38], and it increases also during MS and other neuroinflammatory disorders, suggesting that the microglia response mediated by TREM-2 occurs whenever there is a neuronal injury, so not only in AD [39,40]. Another marker of glial activation is the glial fibrillary acidic protein (GFAP), one of the cytoskeletal filament proteins in astrocytes, which is activated and then released from these cells during neurodegeneration. CSF GFAP levels were reported to inversely correlate with the cognitive function, although an increase of this protein production was found not only in patients affected by AD, but also with FTD and LBD, suggesting its potential use in the prediction of dementia progression [41]. Regarding the microglial and astrocyte marker YKL-40, several studies observed higher levels in the CSF of AD patients as compared with controls [42], and these results have been also confirmed by a recent meta-analysis [43]. CSF YKL-40 increases with disease progression and is positively correlated with biomarkers of neurodegeneration [44]. Some studies have even reported that its levels can predict the progression from cognitively unimpaired to MCI and from MCI to AD dementia [42,45]. Also in the case of YKL-40, its increased levels in CSF are not specific for AD, albeit they are unchanged or even decreased in PD patients without dementia [46].

Finally, several studies also focused on BACE-1 as a possible AD biomarker, but with conflicting results. Most of them showed an increase in activity or protein levels of BACE-1 in AD individuals and also in subjects with MCI who developed AD later, being a good progression marker [47,48,49]. Conversely, other authors reported different results, including no differences in BACE-1 activity between controls, MCI and AD cases, or even decreased CSF levels in AD as compared with healthy controls [50,51]. A summary of levels of CSF biomarkers is reported in Table 1.

**Figure 2:**
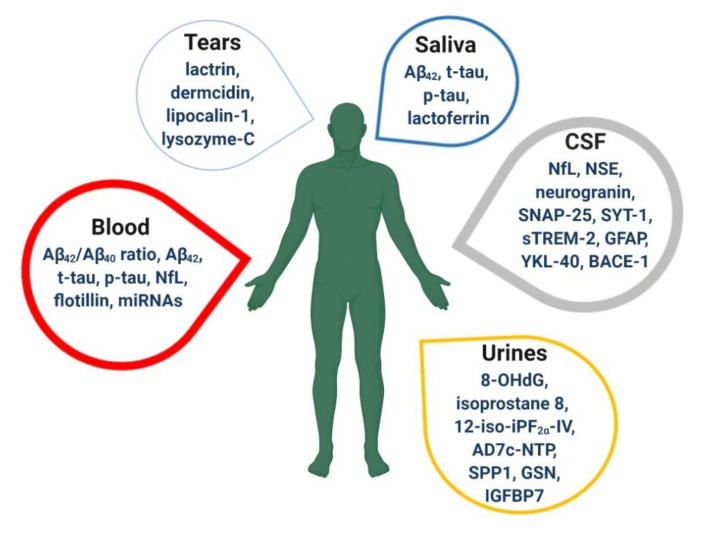
Emerging biomarkers in cerebrospinal fluid (CSF) and alternative biological fluids. For each of them, the thickness of the box is proportional to the number of articles in English searched in Pubmed database on 24 June 2020 using the keywords: “biomarker” AND “Alzheimer’s disease” AND the type of fluid.

ECLIA, electrochemiluminescence immunoassay; ELISA, enzyme-linked immunosorbent assay; MS, mass spectrometry.Overall, the value of these molecules as AD biomarkers has to be validated [52,53]. Moreover, given the fact that CSF collection requires lumbar puncture, there is still a need to discover additional non-invasive, reproducible, reliable, inexpensive and simple to measure biomarkers in alternative biological fluids, e.g., blood, saliva, urine and tears (Figure 2).

### 2.2. Non-Invasive Biomarkers

#### 2.2.1. Blood

As blood is more accessible than CSF, potential biomarkers have largely been studied in this biological fluid [54,55]. Apart from the much less invasive procedure of blood collection as compared to the lumbar puncture, it allows repeated sampling and measurements to monitor the AD progression or to evaluate the efficacy of the newly developed drugs during clinical trials. However, developing blood biomarkers for AD and other brain disorders is still challenging and requires highly sensitive technologies for detection and careful validation work. The blood‒brain barrier (BBB) represents a major issue in finding suitable blood-based biomarkers. Whilst CSF is continuous with the brain extracellular fluid, with a free passage of molecules from the brain to the CSF, the highly selective semipermeable membrane allows entrance only to selected brain-derived proteins, which are typically present at low concentrations [56]. Moreover, some biomarkers related to AD pathology are expressed in non-cerebral tissues and this could confound their measurements in the blood. Finally, the activity of various proteases or protein carriers in blood may participate in degrading or masking the epitopes of a potential biomarker, interfering with its detection and measurement [57].

Several studies on plasma biomarkers have indeed reported inconsistent results, even for the core AD CSF biomarkers like Aβ and tau proteins. While some authors reported high concordance between the levels of these proteins detected in CSF and blood [58], conversely, other studies on Aβ plasma levels demonstrated a lack of correlation between CSF and blood in both Aβ_40_ and Aβ_42_ in AD [43,59,60,61]. This discrepancy may probably be due to the low levels of Aβ peptide in blood or to the analytical sensitivities of enzyme-linked immunosorbent assay (ELISA) [62]. In order to overcome the limits in the detection using traditional ELISA methods, ultrasensitive technologies have been developed with promising results, including single molecule array (Simoa) [62,63], immunoprecipitation coupled with mass-spectrometry (IP-MS) analysis [46] and stable isotope labeling kinetics followed by IP-MS [64]. Using Simoa assay, Aβ_42_ levels and the ratio of Aβ_42_/Aβ_40_ in plasma were shown to correlate with CSF levels and Aβ deposition measured using PET [62,63]. A decreased Aβ_42_/Aβ_40_ ratio was even found in plasma of patients with MCI and preclinical AD [62]. Novel approaches based on MS technology have the advantage of allowing the investigation of various species of Aβ peptides at very low concentrations. Immunoprecipitation coupled with MS was useful to pull down different Aβ fragments, showing a decreased Aβ_42_/Aβ_40_ ratio in plasma with around 90% diagnostic accuracy and a great ability to predict the presence of Aβ plaques in the brain of AD patients detected using Aβ PET imaging [64,65]. Although these assays overcome several comparison and standardization limits, they do not solve the confounding problem with non-cerebral expression of Aβ which is produced by platelets, the primary source of Aβ peptide accounting for 90% of total blood Aβ [66]. Similarly, ultrasensitive techniques have also been used to measure tau protein in blood samples. Several groups reported elevated t-tau levels in the plasma of AD patients as compared with controls, but the overlapping values between these two groups exclude its potential use as a diagnostic tool [67,68,69]. Additionally, plasma t-tau predicted cognitive decline and the risk of dementia [68,69,70]. On the other hand, plasma p-tau achieved promising results, showing higher concentration in AD patients than in control individuals and a strong correlation with CSF p-tau [71].

Advancements in ultrasensitive assays also enabled the accurate measurements of NfL levels, not only in CSF, but also in blood, revealing a tight correlation with its concentration in CSF. Therefore, blood NfL represents a well-replicated and reliable biomarker useful for screening neurodegenerative processes, monitoring disease progression or therapy [72,73,74,75]. Serum or plasma NfL levels were highly increased in AD and MCI cases when compared to controls and in other neurological disorders [16,72,74,75]. Interestingly, CSF and blood NfL levels are related with AD severity markers, including brain atrophy detected using MRI, glucose hypometabolism measured using FDG-PET and cognitive deterioration evaluated using MMSE, suggesting its use as a disease stage biomarker [72,76,77]. More importantly, blood NfL levels were increased in symptomatic familial AD cases but also in pre-symptomatic carriers of AD mutations and correlated with estimated years of symptom onset as well as both cognitive and MRI measures of AD stage [75,78]. Given all these findings, emerging agreements recommend the use of NfL instead of t-tau as an independent marker of neurodegeneration (N) in the AT(N) classification for AD [13].

Recent evidence has pointed out a role of flotillin as a novel AD biomarker [79,80]. This is a membrane-associated protein located in lipid rafts of intra- and extracellular vesicles; therefore, it plays important roles in signal transduction and membrane–protein interactions. Regarding AD pathogenesis, flotillin is involved in several pathological processes, such as APP processing and endocytosis, mitochondrial dysfunction, Aβ-induced neurotoxicity and neuronal apoptosis [80]. A clinical study reported that flotillin levels were decreased in both CSF and serum of AD patients compared with MCI individuals or age-matched non-AD controls. Moreover, flotillin levels in serum negatively correlated with Aβ burden detected using PET, whereas they remained stable with advancing age in healthy controls [79]. Despite the clinical evidence of a diagnostic utility of flotillin in AD being still in its infancy, emerging findings support that it may be used in support of CSF Aβ_42_ and tau levels as well as PET neuroimaging for more efficient and earlier diagnosis for AD [80]. A summary of levels of blood biomarkers is reported in Table 2.

Epigenetics is also of increasing interest in biomarker discovery, with gene regulation by micro(mi)RNAs representing one of the most investigated molecules [81,82,83]. Since miRNAs are dysregulated in the brain, CSF and blood, they may be used as diagnostic and prognostic biomarkers for AD. Several studies identified panels of miRNAs to discriminate AD patients from controls with a good specificity and sensitivity [84,85,86,87]. Most dysregulated miRNAs are associated with molecular mechanisms occurring during AD pathogenesis, such as inflammation, apoptosis, Aβ and tau signaling pathways [88], suggesting them as an alternative and more sensitive approach for detection and management of AD [89]. Although promising, the use of miRNAs in clinical practice still has several limitations, including variations in analytical platforms and different methods of data validation and normalization.

Finally, a number of additional emerging biomarkers have been investigated in various studies aiming to find a potential link with AD pathological processes, including glial activation, inflammation, neurodegeneration, Aβ pathology or degrading enzymes [43,90]. Although most of them displayed significant association with AD in the CSF, the corresponding levels in blood did not reflect such alteration [43].

#### 2.2.2. Saliva

Saliva represents an attractive marker for monitoring diseases as its collection is non-invasive, convenient and cost-effective. Since salivary secretion decreases with aging, it is supposed that changes in biochemical composition may be related to the development of age-associated diseases. Interestingly, it has been reported that proteins from the CNS are excreted into the saliva [91]. Several studies have evaluated salivary Aβ levels but with conflicting results: increased or unaltered levels of Aβ_42_ were found in AD patients as compared with controls [92]. Similarly to Aβ, tau protein was also investigated as a potential salivary biomarker for AD. Specifically, the p-tau/t-tau ratio was shown to be significantly increased in AD patients. Moreover, while data on salivary t-tau are consistently negative, p-tau species in saliva could have greater utility [93,94].

A recent study suggested the use of lactoferrin as a salivary biomarker with high sensitivity and specificity. Lactoferrin, one of the major antimicrobial peptides, is abundantly present in human saliva and shows Aβ-binding properties. Decreased levels of this peptide were detected in patients with both AD and amnestic mild cognitive impairment (aMCI) compared to healthy controls, resulting in 100% sensitivity and specificity. In addition, authors also reported a positive correlation with CSF Aβ_42_, t-tau and the mini-mental state examination (MMSE) [95].

With contrasting results, several other salivary candidate biomarkers have been examined, including mucins, acetylcholinesterase and cortisol; therefore, it is necessary for data to be replicated in larger cohorts or longitudinal studies [96]. Although saliva seems to represent an interesting source of markers, its content may be influenced by the circadian rhythm, flow rate and time of sample collection. Moreover, the levels of biomarkers require normalization and are unstable because of the presence of degradative enzymes (Table 3). 

#### 2.2.3. Urine

In contrast with saliva, the use of urine as a diagnostic biomarker has the advantage of it being easily normalized on measured levels of creatinine, which is physiologically stable. Urine has so far represented a good marker source for the diagnosis and monitoring of renal dysfunctions and urinary tract and prostate cancers. As mentioned above, oxidative stress and oxidative DNA damage play an important role in the early stages of the disorder and are currently being explored for possible biomarkers in AD (Table 4). Specifically, ROS combines with mitochondrial and nuclear DNA to produce 8-hydroxy-2′-deoxyguanosine (8-OHdG), a marker used to monitor cellular dysfunction in urine. It has been reported that AD patients exhibit high urinary levels of 8-OHdG as compared with healthy elderly controls [97]. Another biomarker for oxidative injury is represented by isoprostane 8, 12-iso-iPF_2α_-IV, generated from arachidonic acid peroxidation by free radicals. Elevated levels of isoprostane 8, 12-iso-iPF_2α_-IV were found in all biofluids of AD patients, including CSF, urine and plasma [98].

Increasing evidence supports the use of urinary Alzheimer-associated neuronal thread protein (AD7c-NTP) as a potential candidate for AD early diagnosis. The transmembrane phosphoprotein AD7c-NTP co-localizes with NFTs and is positively associated with phosphorylated tau accumulation in CSF from AD patients. Several studies reported increased AD7c-NTP levels in CSF and urine in the early course of neurodegeneration in AD, which is positively associated with the disease severity. Moreover, a meta-analysis suggested a possible use of urinary AD7c-NTP in the early diagnosis of AD [99].

A recent study coupling computational and experimental methods revealed three differentially expressed proteins in the urine of AD patients: SPP1 (secreted phosphoprotein 1), GSN (gelsolin) and IGFBP7 (insulin-like growth factor-binding protein-7). Interestingly, all of them have already been reported to play an important role in AD pathogenesis. In AD models, SSP1 is involved in modulating the macrophage immunological profile towards a better capacity in mediating Aβ clearance. GSN binds to Aβ, solubilizing its existed fibrils or inhibiting Aβ fibrillization in the brain, whereas IGFBP7 contributes to brain cell homeostasis and is a critical mediator of memory function [100].

#### 2.2.4. Tears

Tear samples have been already suggested as an excellent biomarker candidate, providing information not only on pathological conditions affecting the ocular system, but also on systemic pathophysiological processes [101,102]. Interestingly, Aβ plaques were found in the retina and lens of AD patients, as well as changes in the retinal morphology and vasculature, resulting in an impaired visual performance [103]. Thus, an alteration of the eye microenvironment may be reflected at the level of tear proteins. In a recent pilot study using quantitative proteomics techniques, the authors found a change of tear flow rate, total tear protein concentration and composition of the chemical barrier specific to AD (Table 5). Moreover, a very high accuracy in discriminating AD patients from healthy donor controls has been reached by the combination of a panel of proteins, including the extracellular glycoprotein lacritin, dermcidin, lipocalin-1 and lysozyme-C, which are mostly involved in the inflammatory processes and defense against pathogens [104].

## 3. Emerging AD “Dry” Biomarkers: Structural and Functional Techniques

**Figure 3:**
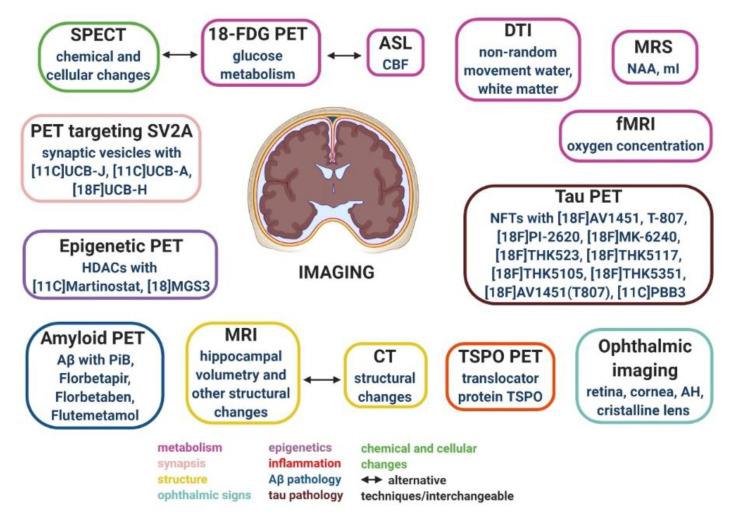
Neuroimaging techniques and their validated molecular targets for studying morphological and functional changes occurring in AD.

Several brain-imaging and neurophysiological techniques could be used for studying morphological and functional changes occurring in AD [105]. Quantitative electroencephalography (EEG) and other neurophysiological biomarkers like event-related potentials and transcranial magnetic stimulation (TMS) have been used to predict MCI conversion to AD and for dementia differential diagnosis, but more research should examine their sensitivity and specificity for diagnostic purposes [106,107,108]. Morpho-functional imaging studies have been used according to the NIA-AA guidelines and have the advantage over fluid biomarkers to provide crucial disease-staging information, as imaging can distinguish the different phases of the disease both temporally and anatomically [12]. We could subdivide AD imaging techniques into two main categories: structural and functional imaging (Figure 3).

### 3.1. Structural Imaging

Structurally, AD patients show characteristic patterns of atrophy involving several structures of the medial temporal lobe (MTL) including the hippocampi, amygdala, the cingulate cortex, parahippocampal gyrus and the entorhinal cortex [109,110,111,112]. The two main techniques providing structural data on the brain of AD patients are computed tomography (CT) and MRI. Several guidelines suggest using one of them for evaluation of patients presenting with a cognitive/dementia syndrome (CDS) in the clinical setting [110]. Diffuse cerebral atrophy, with enlargement of the cortical sulci and increased size of ventricles are easily detectable in AD using both CT and MRI. However, despite the fact the CT imaging techniques are preferable to MRI in non-collaborative patients, where you need speed, for a minor cost, and also higher availability in developing nations, quantitative measurements are limited using CT. Moreover, CT is not sensitive for early changes, and so it is not considered as a standard biomarker for early diagnosis of AD.

#### 3.1.1. MRI

The MRI technique is the most used in AD diagnosis due to its high spatial resolution, which allows the difference between two arbitrarily similar but not identical tissues and multiparametric acquisitions to be distinguished [113,114]. MRI does not involve X-rays or the use of ionizing radiation, it is non-invasive and has no significant adverse health effects. Moreover, this technique has revealed the ability to define a spectrum of changes related to vascular pathologies and white matter diseases in the brain and permits detection of microstructural and biochemically changes, thanks to diffusion tensor imaging (DTI) and MR spectroscopy (MRS) techniques, respectively, and functional studies as well.

Since 2008, Whitwell and colleagues, studying AD patients, demonstrated the existence of a correlation between MRI volumetric measurements of MTL and, in particular, the hippocampus and NFT accumulation in the lobes [115]. Hence, the measure of hippocampal volume has become a well-established biomarker for AD diagnosis and follow-up [116,117,118]. Some MRI studies have also shown a correlation between the extent of hippocampal and entorhinal volume decline and the increasing age predicted performance on memory tasks [119,120,121,122]. However, the underlying pathological mechanism is not clear. In particular, it could not be inferred that these changes are actually the result of cell loss with age, because it could also be plausible that they really are secondary to synaptic and dendritic loss.

Hippocampal volumetry has shown an important limit, which is the absence of great sensitivity during the prodromal stage of the disease. Therefore, new MR-derived biomarkers are needed with a higher sensitivity compared to whole hippocampal volumetry at an early disease stage. Moreover, clinical routine has shown that it is very challenging to quantify the degree of atrophy for each single case, while it is evident based on several reports in the literature that in large research studies based on group level analysis, an AD “signature” atrophy could be easily detected. As stated above, the evaluation of whole hippocampal volume was not a well-suited biomarker during the pre-clinical AD stage. Several papers reported contradictory findings, potentially due to the use of different approaches in performing the analyses [123]. This could be related to the fact that the manual volumetric technique is a very challenging and time-consuming approach also requiring an excellent working knowledge of neuroanatomy as well as good skill in delineating regions of interest (ROI). Several studies on hippocampus volumetry in middle-aged adults below the age of 55 years reported a contradictory pattern. In particular, while Okonkwo and colleagues found characteristic left posterior hippocampal atrophy [124] and O’Dwyer et al. reported reduced right hippocampal volume in young APOE carriers [125]; others were not able to find differences between the high- and low-risk groups [119]. To overcome difficulties in whole hippocampal volumetry analysis, more recent studies have used segmentation techniques to quantify volumes of each functionally specialized subfield of the hippocampus, which has discrete histological characteristics. This approach demonstrated a higher sensitivity in capturing subtle atrophy patterns compared to whole hippocampal volumetry [126]. A recent work suggested that surface deflections across all hippocampal subfields (CA1 lateral zone, dentate gyrus/CA2-4 superior zone and subiculum inferior medial zone) could be used as biomarkers to differentiate early AD patients from non-demented controls [127].

Currently, the most recurrent volumetry assessment tools and image analysis methods for AD include manual tracing for image processing analysis and visualization; automated Voxel-based morphometry (VBM) for investigation of focal differences in brain anatomy (it allows healthy controls and patients to be distinguished based on the volume of brain and ROI) [128,129]; individual brain atlases using statistical parametric mapping (IBASPM) to automatically create segments of cerebral structures and compute the volume of gross anatomical structures to distinguish an AD group from a normal control group; the insight segmentation and registration toolkit-SNake automatic partitioning (ITK-SNAP), which is a semi-automated 3D brain segmentation technique; and FreeSurfer, which automatically identifies and measures brain regions detecting hippocampal atrophy in patients and providing information about the shape, position or volume of brain tissue.

FreeSurfer software is one of the two main methods for hippocampal segmentation. The other one is automatic segmentation of hippocampal subfields (ASHS). Both support multispectral segmentation relying on atlases based on histological information and high-resolution ex vivo MRI scans. However, the use of the segmentation methods has not yet demonstrated its validity in the study of healthy adults at risk of AD as the results have been inconclusive [130]. Very recently, an MRI biomarker for in vivo AD diagnosis based on the use of FreeSurfer and a supervised machine learning approach was reported [131]. Based on an individual’s pattern of brain atrophy, a continuous AD score is assigned which measures the similarity with brain atrophy patterns seen in clinical cases of AD [131].

Besides hippocampal volume, structural MRI also allows cortical thinning to be studied in the entorhinal cortex (EC), another biomarker identified as a highly sensitive measure of structural change both in MCI and AD [132]. The decline in EC thickness occurs earlier and could be used as a predictive marker of hippocampus volume decline.

Structural MRI methodological limitations are, in particular, susceptibility to movement, differences in spatial resolution across scans that make it difficult to perform a comparison and difficulties in determining the real neural source of volume or thickness loss (cell loss vs. dendritic and synaptic loss) in the absence of high-resolution scanning. This is a concern due to the fact that high resolution and post-processing studies are not feasible in many institutions.

#### 3.1.2. Diffusion Tensor Imaging (DTI) and Biomarker of White Matter Integrity

DTI is an advanced MRI which measures non-random movement of water molecules and also tracks the fibers of tracts within the brain. It has been used to study the microstructural features of white matter [133]. In AD, these studies showed fiber alteration in the temporal and frontal lobes and also corpus callosum and posterior cingulate gyrus [134] with a posterior to anterior gradient [135]. A modest diagnostic power in discriminating AD from controls has been reported in a meta-analysis using DTI measurements of limbic regions [136].

DTI could be used in clinical trials to monitor response to disease modifying drugs or as an indicator of disease progression owing to the fact that functional modifications are detectable prior to structural abnormalities. However, DTI use in AD diagnosis is at the early stages of evaluation and requires further studies. DTI has a number of technical limits that reduce its utility in clinical settings: there is significant variability of DTI-based diffusion metrics between MRI scanners, and traditional approaches cannot resolve intra-voxel complexities such as fiber bending, crossing and twisting [137]. The latter limits may be overcome in the future using high angular resolution diffusion imaging (HARDI), which provides correct information to model diffusion with an orientation distribution function (ODF), a more versatile diffusion representation that captures multiple orientations in a voxel [138].

#### 3.1.3. Proton Magnetic Resonance Spectroscopy (MRS) and Metabolic Markers of AD

MRS is an imaging method for which three decades of research indicate a potential role as a biochemical imaging marker in AD [139]. MRS aims to measure chemical concentration in the brain. The most common compounds analyzed in MRS are represented by N-acetylaspartate (NAA), choline (Chol), myoinositol (mI), glutamate plus glutamine (Glx) and creatine (Cr). The last one is generally considered as an internal reference due to it having no significant changes in different conditions, and MRS is then reported as a ratio of the tested metabolite over Cr [140].

Since 1992, a reduction in NAA neuronal metabolite has been demonstrated using MRS performed on autopsy brain samples of AD patients in respect to levels detected in healthy controls. The observed decrease of NAA was also demonstrated to correlate with the amount of tangle and plaque in the brain [141]. The decrease in NAA or NAA/Cr has been then considered as a marker of neuroaxonal density and viability [142]. AD patients show reduced NAA/Cr ratio in posterior cingulate voxels [143] and in the medial temporal lobe [144] and hippocampi [145] compared to controls. The same decrement is not detectable in MCI patients because these areas are not yet involved in the neurodegeneration [142].

Myoinositol, a glial marker, was also analyzed in AD patients through MRS and its increase was detected in several brain regions. This is thought to be an early event in the course of AD pathology preceding NAA reduction [142].

Choline has also been analyzed in AD as a possible biomarker in MRS studies. However, contrasting data have been reported [145,146,147,148,149,150,151]. Inconsistent changes were also reported using MRS testing GLX [143,152]. The advantages of MRS compared to other functional imaging techniques are that it is more widely available, much less expensive, has no radiation risk and can be added to the structural MRI sequences to extract useful information to help diagnosis. MRS can be used as a follow-up imaging tool in therapeutic trials correlating the level of metabolites and pathological changes, or it can be used as a tool to predict cognitive impairment in combination with other biomarkers.

However, MRS is still not routinely used clinically because further research is required to standardize the techniques, to compare the results of MRS with other functional biomarkers and to better understand the pathological substrates for metabolite abnormalities.

### 3.2. Functional Imaging

Functional imaging includes recently developed techniques not yet well applied in worldwide routine clinical settings. In particular, these techniques include PET with different tracers, single photon emission computed tomography (SPECT) and advanced MRI techniques such as functional MRI (fMRI) and arterial spin labeling (ASL).

#### 3.2.1. Single Photon Emission Computed Tomography (SPECT) and Perfusion Imaging

Another molecular technique useful in discriminating individuals with AD is SPECT, which is able to detect both chemical and cellular changes linked to a disease through the use of highly targeted radiotracers [153]. SPECT evaluates cerebral perfusion and shows good correlation with metabolic changes. Perfusion hexamethylpropyleneamine oxime (HMPAO)-SPECT is seen as an alternative to 18F-FDG PET even though SPECT has lower sensitivity and specificity than FDG for the diagnosis of AD [154]. Abnormalities on perfusion SPECT in AD are represented by hypoperfusions commonly affecting temporoparietal areas in a bilateral distribution, with the posterior cingulate and medial temporal areas particularly affected in AD and the sensory motor cortices, including the cerebellum, largely spared [155]. Studies of the accuracy of SPECT for diagnosing AD report sensitivities of 65–85% and specificities (for other dementias) of 72–87% [156]. SPECT has the advantages of being more widely available, cheaper than FDG PET and better tolerated by the patient. (HMPAO)-SPECT was demonstrated to have less diagnostic accuracy than FDG PET; thus, it could be helpful for the dementia/no-dementia comparison but not in differentiating AD from DLB [154].

#### 3.2.2. PET

PET scanning is a well-established molecular imaging technique resulting in 3D images of what is happening in a patient’s brain at the molecular and cellular level [157]. It is very accurate at diagnosing AD and differentiating it from other dementias. Consensus agreements suggest the use of PET biomarkers in patients suspected to be affected by AD without meeting NIA-AA criteria for dementia and with a well-defined clinical and cognitive profile. In these cases, PET imaging might support or exclude the clinically suspected etiology avoiding the unnecessary use of costly and potentially harmful treatments and allowing a rapid and accurate diagnosis. This has important positive impacts on the patient, his family and society [158].

Several tracers with different specificity have been developed to study AD patterns in different stages of severity. In particular, ligands were designed to study Aβ deposition, synaptic dysfunction (mainly cortical hypometabolism) or tau fibrillary tangle deposition.

In the following sections we will briefly describe the most used PET ligands having diagnostic and prognostic significance in AD.

##### PET and Metabolic Biomarkers

18F-FDG PET measures the glucose metabolism in different regions of the brain and represents a metabolic marker in early diagnosis and preclinical detection of dementia, metabolic changes usually becoming impaired before the appearance of detectable structural changes in the brain. This technique, which also points to vascular deficits and impairment in the blood‒brain barrier frequently found in AD, can be used when the clinical diagnosis still remains in doubt to identify the causes of dementia and to have a differential diagnosis. Hypometabolism in 18F-FDG PET is considered a biomarker of neuronal dysfunction, neurodegeneration and synaptic disease.

Cerebral glucose hypometabolism has been consistently demonstrated in the medial temporal lobe, posterior cingulate gyrus, parieto-temporal regions and/or frontal cortices of typical AD patients, while it has been detected to be moderate in the basal ganglia, cerebellum, thalamus and sensorimotor and visual cortices [159,160]. The observed metabolic changes associated with neocortical dysfunctions may predict successive atrophy and suggest that a conversion of MCI to AD could occur within several years [161,162]. Moreover, a correlation was reported between the performance of patients on a cognitive test and the hypometabolic patterns during disease progress [163] and clinical symptoms of cognitive impairment [164].

The use of FDG PET instead of structural MRI is suggested in rapidly converting early MCI individuals, while structural MRI may outperform FDG PET in late MCI subjects [165,166]. The FDG PET technique sensitivity as well as specificity are very high (>90%) in discriminating healthy elderly individuals from AD, while the specificity was reported to be reduced (78%) in differential diagnosis of AD and other dementia [167]. The use of FDG PET is limited by the fact that it is an expensive technique that also requires exposure to ionizing radiation.

Like in the case of MRI reported above, PET images can also be assessed in different way. One of them is visual inspection, but the efficacy of this largely depends on the experience of the reader, and it might be very challenging in the study of patients with mild disease due to a lack of clear cut-off between normal and abnormal values. In these cases, the use of standardized uptake value ratio (SUVr) quantification is usually suggested instead of visual inspection. SUVr utilizes static imaging and is evaluated in respect to a region without altered metabolism or with mildly affected metabolism in AD [168].

##### PET and Amyloid Imaging Biomarkers

In the past decades, the possibility of direct imaging of AD brain lesions and in particular of the presence of Aβ aggregates has been investigated and several tracers with different half-lives and affinities to plaques have been engineered to be used for this purpose [169].

A lot of studies on Aβ deposition in AD are now available linking this pathological finding with several other correlates such as aberrant entorhinal activity among cognitively normal older adults and cortical thinning in frontoparietal regions [170,171]. Moreover, it was reported that Aβ deposition may predict tau deposition during aging [172]. In fact, amyloid imaging studies highlighted that the deposition onset usually starts in the posterior cingulate, restrosplenial cortex and precuneus regions [111]. These brain regions are interconnected with the MTL, which, on the contrary, is a site for early tau aggregation, and this suggests the possibility of an influence of anatomical and functional connectivity between all these brain areas in the progression of the disease.

Amyloid imaging uses PET technology to acquire images of the brain in order to display foci of abnormal amyloid accumulation after an injection of a radiolabelled ligand specifically targeting amyloid aggregates. Among tracers, the first ligand developed was the Pittsburg compound B (PiB), a fluorescent derivative of the amyloid-binding histological dye, thioflavin-T [173]. Being the first reported, it is also the best characterized amyloid tracer. It was demonstrated that it can bind selectively with high affinity to fibrillar Aβ aggregates but not to amorphous Aβ deposits or NFTs [170,171]. In fact, studies reported in the literature demonstrate a high affinity of PiB to frontal, temporo-parietal and posterior cingulate cortices, while minimal binding was reported in the cerebellum and other regions with typical low density of Aβ plaques [174,175].

The use of the PiB tracer has a number of limitations including a very short life (around 20 min), the need of an on-site cyclotron and 11C radiochemistry expertise, the fact that it has also displayed retention in the brain of nondemented people [176] and high affinity for vascular deposits that could also be observed in non-AD conditions [177] and, finally, it has low ability to detect soluble oligomeric Aβ conformations considered to be highly pathogenic [178].

Due to these limitations in the use of PiB, newer fluorinated tracers with longer half-lives have been developed and are increasingly used. They include Florbetapir F 18-Florbetapir F 18 (18F-AV-45), Florbetaben (BAY 94-9172) and 18F Flutemetamol (Flute), which is structurally identical to PIB except for one fluorine atom in the place of a carbon atom [179].

Despite the common agreement in considering amyloid PET as the most specific and sensitive biomarker for AD, its usefulness in clinical settings is under evaluation [180]. It is suggested that this technique should be used only in dementia expert centers to confirm AD diagnosis in atypical cases or in the differential diagnosis between amyloid-associated dementia and non-amyloid pathology. Notably, this technique is not able to discriminate among stages of dementia progression and has the caveat in preclinical AD studies that 10–30% of cognitively normal individuals can have positive amyloid PET [176,181].

##### PET and Biomarkers of Synaptic Damage or Loss

The existence of a strong association of synapses and AD pathophysiology is well known. Thus, biomarkers of synapse damage or loss as proxies for synaptic and cognitive function in AD have been studied. Very recently, new PET ligands ([11C]UCB-J, [11C]UCB-A and [18F]UCB-H) have been developed labelling the synaptic vesicle glycoprotein 2A (SV2A) and allowing synapses to be visualized in the living brain [182,183,184]. In particular, the use in PET scanning of [11C]UCB-J demonstrated a reduction of approximately 40% of SV2A signal in the hippocampus of AD cases in respect to cognitively healthy aged cases [185]. Although very few studies have been reported using this approach to measure synapse loss longitudinally in AD, it appears that a direct measure of synapse density is a very promising biomarker to be used probably in combination with one of the previously reported biomarkers in CSF or MRI or FDG PET imaging biomarkers.

##### PET and Tau Biomarkers

The presence of NFTs, p-tau protein aggregates, in AD suggests the use of tau imaging as a surrogate marker to predict cognitive decline or disease progression in AD.

Since 2002, selective tau PET tracers have been developed. The first tracers were quinolone and benzimidazole derivatives with affinity to the PHF among tau aggregates. This target was optimal for the use of the ligand because it co-occurs with Aβ. This created an additional challenge as the ligand could bind both PHF and Aβ. However, a 25-fold selectivity for PHF over Aβ was achieved [186].

Among the first generation tau selective PET tracers, the two mostly studied were [18F]AV1451 and T-807. Beside the advantage of being able to replicate features of the Braak histopathological changes, these tracers had two important limitations: they had increased striatal retention and they had off-target binding to monoamine oxidase A/B [187]. Then, they were quickly substituted by a second generation of new targets without off-target binding such as [18F]PI-2620 and [18F]MK-6240 [188]. Through the use of these new ligands, a specific tau PET signature was reported in AD patients starting from the transentorhinal/entorhinal cortex to the hippocampus and then extending to the rest of the temporal lobe and neocortical regions [189]. This stepwise pattern typical in AD is not commonly detected in cognitively normal individuals and could be then be used in staging the disease.

In more recent years, additional different tau selective PET tracers have been engineered to be used for human studies: [18F]THK523, [18F]THK5117, [18F]THK5105, [18F]THK5351, [18F]AV1451(T807) and [11C]PBB3 [190]. Despite the reported results being promising up to now, the characteristics of tau intracellular aggregates, which are subjected to conformational changes and coexist with a high concentration of amyloid deposition, complicate the use of tau imaging for clinical use. For future use of this approach in the clinical setting, tau PET tracers must be developed with a higher selectivity and affinity to tau compared to Aβ and they must be able to cross the blood-brain barrier without being metabolized [186].

##### Novel PET Approaches: Inflammatory and Epigenetic Biomarkers

New imaging approaches to study AD are being developed. One of them is TSPO-PET, a PET technique performed using a specific ligand for the translocator protein TSPO (previously named peripheral benzodiazepine receptor), a mitochondrial membrane protein involved in several functions relevant to neurodegeneration and upregulated in neuroinflammation. Increased TSPO expression was detected in AD animal models, and it was demonstrated to correctly overlap with brain pathological areas as well as with regions of increased immunohistochemical staining of TSPO [191]. Moreover, the study of TSPO PET signals in preclinical trials of novel therapeutic AD models suggested that it could be used as a biomarker to monitor treatment progress in clinical trials [192]. However, new TSPO PET radioligands are needed due to the fact that those currently available lack high affinity to a prevalent polymorphic form of TSPO (A147T) [193].

Another novel PET approach to AD is imaging epigenetics ([11C]Martinostat PET). Epigenetics consists of several newly detected mechanisms involved in regulating gene expression. The two main epigenetic mechanisms, both modifying chromatin structure, are DNA methylation and histone acetylation. The latter, and in particular the involved histone deacetylase (HDACs), is the mechanism targeted by [11C]Martinostat PET. In fact, the Martinostat radiotracer was designed with high specific binding of a subset of class I HDAC enzymes (isoforms 1, 2 and 3) and thus is able to image HDAC density with favorable kinetics and high affinity [194]. The rationale for the use of this PET approach for studying AD resides in the observation that epigenetics has a role in the dynamic process of learning and memory and that it is altered by AD pathology. However, there is not a clear causal-consequence correlation between epigenetics and AD owing to the observation that some epigenetic changes arise after AD onset [195,196] while other changes arise before the disease presentation [197]. HDAC expression was reported to be lowest in the amygdala and hippocampus among gray matter regions in healthy adults [198]. A new epigenetic PET tracer has been recently developed (the fluorinated variant of [18F]MGS3) showing specific binding, comparable brain uptake and regional distribution to [11C]Martinostat, but due to technical limits in the radiosynthesis process no validation has been done [199]. Epigenetic imaging is still a new technique and will be further improved, but it might become useful in analyzing gene regulatory processes underlying AD and might be considered in the future as a biomarker for AD.

#### 3.2.3. fMRI and Metabolic Markers

Functional MRI (fMRI) consists of the acquisition of brain images during a specific brain activity and in a basal state, and it measures the oxygen concentration of certain specific brain areas corresponding to particular stimuli or cognitive tasks [200,201]. fMRI studies showed a decrease in coordinated activity of AD patients in the hippocampus and inferior parietal lobes and cingulate cortex compared with healthy controls [202,203]. fMRI can be performed in resting state to study synaptic integrity and circuit connectivity or be performed in task-activated state to study reduced inhibition and hippocampal hyperactivity.

In preclinical AD, resting state fMRI (rsfMRI) has been linked to metabolic changes and precedes neurodegeneration (reviewed by [204]). Most rsfMRI analyses focused on the default mode network (DMN) that involves the medial prefrontal cortex, posterior cingulate cortex, precuneus, anterior cingulate cortex, parietal cortex, and the medial temporal lobe, including the hippocampus. These studies demonstrated widespread changes in DMN connectivity in MCI and AD [205,206,207,208]. Other studies pointed also to a disruption in the connectivity within the MTL (between the entorhinal cortex and the dentate and CA3 regions of the hippocampus) [209] and in other networks [210]. Functional connectivity is then considered an early marker of synaptic pathology due to isolation of the hippocampus from its cortical input.

Task-activated fMRI was also used in several studies and resulted in a number of observations: mild AD cases have a decrease in hippocampal activity similar to more impaired MCI patients [211]; the extent of hippocampal hyperactivation at baseline predicted cognitive decline as measured by the CDR-SB scores over four years after scanning [212]; the hyperactivation is specific to the DG/CA3 subregions of the hippocampus [213].

#### 3.2.4. Arterial Spin Labelling ASL and Metabolic Markers

ASL perfusion MRI is a non-invasive technique for measuring cerebral blood flow (CBF) using magnetically labelled arterial blood water protons as endogenous tracers [214]. This technique has been studied to evaluate the possibility of using it instead of FDG PET in AD diagnosis, ASL being cheaper, with no radiation risk and more widely available. These studies revealed a good concordance of ASL and FDG PET results with comparable diagnostic accuracy [215]. Given the possibility of adding ASL to routine structural MRI protocols without additional cost, it appears a promising method for classifying the degree of neurodegeneration in individuals with prodromal AD. However, some limitations that are still present (e.g., the sensitivity to blood velocity and arterial transit time, the interferences occurring in the presence of steno-occlusive disease or other cerebrovascular pathology and the sensibility to head motion) are still reducing its clinical use [216].

## 4. Ocular Biomarkers of AD

Very recently, several groups started to consider the ocular examination as new non-invasive tool for AD screening and diagnosis. The rationale for the focus on eyes is that they have an origin that is similar to the brain’s and that neurons in the cerebral cortex are similar to those in the retina [217,218,219]. Changes in the neural and non-neural ocular tissues have been reported in AD [220]. These include accumulation of Aβ and degeneration of retinal axonal and neural tissue.

In the cornea of AD patients, several abnormalities have been reported including corneal lattice dystrophies, degeneration of the corneal sub-basal nerve plexus, corneal nerve fiber pathology and decreased corneal sensation [221,222,223]. The detection in the aqueous humor (AH) of Aβ in patients with pseudoexfoliation syndrome and glaucoma suggested a link between these conditions and AD and the usefulness of searching for APP and Aβ in the AH as a biomarker of the disease [224]. Studies are needed to check whether AD has a direct or indirect neurotoxicity on the corneal nerve plexus. A correlation between AD and changes in the crystalline lens was also reported in a number of studies, making it a promising biomarker tissue for disease diagnosis. In particular, patients show a progressive loss in the transparency and cataractous changes of the otherwise transparent crystalline human lens [102].

Changes have also been reported in several aspects of the retina. In particular, retinal vessels of AD patients show reduced caliber of retinal veins, reduced arteriolar and venular fractal dimensions, smaller, more tortuous and sparse retinal vessels and reduced flow of blood [225,226]. Moreover, a thinning of the retinal nerve fiber layer and the degeneration of the retinal ganglion cell were also reported in AD patients [227,228]. An optical retinal imaging platform has also been developed to detect Aβ plaques in retina in AD [229].

The use of ocular biomarkers will be very helpful in the future in diagnosing AD at an early stage or to follow-up the patient during treatment with therapeutic agents. However, their implementation to the population for screening purpose remains a challenge.

## 5. Conclusions

Despite the high diagnostic accuracy of the established CSF biomarkers for AD, Aβ42, t-tau and p-tau, several other candidates in alternative non-invasive biological fluids have been recently investigated for their potential clinical use in support of early AD diagnosis and prognosis (Table 6). Advancements in ultrasensitive assays enabled the precise measurements of analytes and decreased the intra- and inter-laboratory variation, helping to identify novel candidate biomarkers as well as to support harmonization efforts for the core biomarkers for AD. An increasing number of molecules have been identified so far and the highest potential was reached for NfL in both CSF and blood, lactoferrin in saliva and Aβ_42_ and p-tau in plasma. Nevertheless, there are several issues related to most of the presented potential AD fluidic biomarkers: they often come from a single study or there is significant inconsistency in the results from different studies. Additionally, pre-analytical sample processing should be standardized, analytical methods validated and the impact of other factors, such as age, presence of comorbidities and disease diagnosis/stage, must be thoroughly evaluated. Advanced imaging techniques partially overcome these limitations, allowing the identification of AD-related structural and functional biomarkers (Table 7). They provide easily interpretable data for determining AD stage, giving a very high accuracy for disease diagnosis. The useful in clinical settings of several imaging approaches and novel biomarkers is still under evaluation. In fact, the use of each technique shows both limits and advantages and additional studies are required to define which one could be the best. Moreover, brain imaging is expensive, time-consuming and the equipment is not always available, thus limiting access to most of population. In this context, the recent developed biosensors are emerging as promising alternatives for rapid, low-cost and simple diagnosis for AD, even in the early stages. With high sensitivity and selectivity, they represent excellent analytical tools which have applications in detecting AD biomarkers, being applicable to easily sampled biological fluids, including blood, urine and tears.

It is now commonly ascertained that the best way to adequately make a diagnosis of AD and the staging of the disease progress is to combine two or more of the above-reported biomarkers, particularly mixing together fluidic molecular analysis and imaging studies. The choice of which specific biomarkers and techniques to combine depends on the case under evaluation. In particular, different combinations will be properly used for studying AD patients at different stages of the disease or in monitoring the course of AD during drug treatment and clinical trials or finally in differential diagnosis. This is due to the fact that each biomarker and technique has different efficacy in the diagnosis, prognosis and staging of the disease. However, great attention still needs to be paid to several aspects of study design, sample collection, sample measurement and data analysis, and international collaboration to standardize assays and study protocols, as well as to recruit sufficiently large cohorts, will facilitate future biomarker discovery and development.

Studies should still be addressed towards the identification of a non-invasive biomarker for predicting AD before the onset of symptoms. Despite extensive research worldwide, no diagnostic method is currently available for pre-clinical AD and the existing treatments are only symptomatic.

## Figures and Tables

**Table 1:** Changes in levels of CSF biomarkers in Alzheimer’s disease (AD) patients and controls.

Biomarker	AD	Controls	AD/Controls	Technique	Reference
NfL (pg/mL)	1574.04–2827.96	995.41–2016.59	↑	ELISA	[16]
NSE (ng/mL)	15.63–20.60	5.98–10.94	↑	ECLIA	[19]
neurogranin (pg/mL)	349–744	161–453	↑	ELISA	[26]
SNAP-25 (ng/mL)	22–38	18–20	↑	MS	[28]
SYT-1 pM	131.7–449.7	166.9–309.7	↑	ELISA	[30]
sTREM-2 (pg/mL)	172.5–305.4	131.0–240.7	↑	ELISA	[34]
GFAP (ng/mL)	1.77–4.26	1.31–3.21	↑	ELISA	[41]
YKL-40 (ng/mL)	400–422	254–293	↑	ELISA	[20,42]
BACE-1 activity (pM)	30–43	23–42	↑≅	ELISA	[47,50]

**Table 2:** Changes in levels of blood biomarkers in AD patients and controls.

Biomarker	AD	Controls	AD/Controls	Technique	Reference
Aβ_42_/Aβ_40_ ratio	0.04–0.08	0.05–0.1	↓	Simoa	[62]
Aβ_42_ (pg/mL)	5.9–20.5	14.4–24.8	↓	Simoa	[62]
t-tau (pg/mL)	2.61–4.73	1.98–3.50	↑	Simoa	[67]
p-tau (pg/mL)	>0.0921		↑	ELISA	[71]
NfL (ng/L)	24.1–77.9	13.3–56.1	↑	ELISA	[72]
flotillin (% over controls)	−30		↓	WB	[79]

Simoa, single molecule array; ELISA, enzyme-linked immunosorbent assay; WB, Western blotting.

**Table 3:** Changes in levels of salivary biomarkers in AD patients and controls.

Biomarker	AD	Controls	AD/Controls	Technique	Reference
Aβ_42_ (pg/mL)	41–51.7	21.1–29	↑	ELISA	[92]
t-tau (pg/mL)	~11.5–14.5	~14–17	≅	ELISA	[93]
p-tau (pg/mL)	~90–140	~85–105	↑	ELISA	[93]
p-tau/t-tau ratio	~9–12	~6.5–7.5	↑	ELISA	[93]
lactoferrin (μg/mL)	3.67–5.89	8.28–12.20	↓	ELISA	[95]

ELISA, enzyme-linked immunosorbent assay.

**Table 4:** Changes in levels of urinary biomarkers in AD patients and controls.

Biomarker	AD	Controls	AD/Controls	Technique	Reference
8-OHdG (nmol/L)	99–159	16.5–33.1	↑	HPLC	[97]
isoprostane 8, 12-iso-iPF_2__α_-IV (ng/mg creatinine)	4.51–5.35	1.60–1.94	↑	MS	[98]
AD7c-NTP (μg/mL)	>22		↑	ELISA	[99]
SPP1 (ng/mg total protein)	~8–10	~12–18	↑	ELISA	[100]
GSN (pg/mg total protein)	~1300–1800	~1000–1200	↑	ELISA	[100]
IGFBP7 (pg/mg total protein)	~6–8	~4.8–5.2	↑	ELISA	[100]

HPLC, high performance liquid chromatography; MS, mass spectrometry; ELISA, enzyme-linked immunosorbent assay.

**Table 5:** Changes in levels of biomarkers in tears from AD patients and controls.

Biomarker (Log2 Fold Change Over Controls)	AD	AD/Controls	Technique	Reference
extracellular glycoprotein lacritin	−2.04	↓	MS	[104]
dermcidin	0.85	↑	MS	[104]
lipocalin-1	−0.76	↓	MS	[104]
lysozyme-C	−1.11	↓	MS	[104]

MS, mass spectrometry.

**Table 6:** Main advantages and disadvantages of biological fluid biomarkers for Alzheimer’s disease.

Fluid	Advantages	Disadvantages
CSF	Reliability	Invasiveness
blood	accessibility, reproducibility	need for validation, low protein concentration, difficulty of detection due to the presence of proteases or protein carriers
saliva	accessibility, cost-effectiveness	requirement of normalization, influence by circadian rhythm, flow rate and time of sample collection
urine	ease of normalization on creatinine	need of validation in larger and longitudinal studies
tears	information on systemic pathophysiological processes	need for validation on large scale populations

**Table 7:** Summary of characteristics of main “dry” biomarkers for Alzheimer’s disease.

Imaging Biomarker	Type of Information	Early Changes Predictive for AD	Limits
***MRI Imaging***	N		need patients’ collaboration time-consuming analyses
Morphometric technique Hippocampal segmentation DTI		most studied, good predictive value promising tool, more studies are needed	
Functional technique fMRI ALS		promising tool, more studies are needed more studies are needed	
***Nuclear Medicine Imaging***			
Perfusion SPECT PET Imaging FDG PET	N		invasiveness, ionizing radiation invasiveness, ionizing radiation expansive,
Amyloid PET Tau PET	N P P		invasiveness, ionizing radiation expansive low specificity small studies

D, Aβ dysfunction; N, neurodegeneration; P, pathognomonic; AD, pathological deposition of amyloid or fibrillary tau.
